# Supplementary material for: Development and structural basis of a two-MAb cocktail for treating SARS-CoV-2 infections
Source: Nat Commun. 2021 Jan 11;12:264. doi: 10.1038/s41467-020-20465-w (PMC7801428; doi:10.1038/s41467-020-20465-w)
Supplement: Supplementary file 1 — Supplementary Information [file 41467_2020_20465_MOESM1_ESM.pdf]

Supplementary Information for:

Development and structural basis of a two-MAb cocktail for treating SARS-CoV-2 infections

Chao Zhang[1, #], Yifan Wang[2, #], Yuanfei Zhu[3, #], Caixuan Liu[2, #], Chenjian Gu[3, #], Shiqi Xu[1, #], Yalei Wang[1], Yu Zhou[1], Yanxing Wang[2, 4], Wenyu Han[2], Xiaoyu Hong[2], Yong Yang[1], Xueyang Zhang[1], Tingfeng Wang[1], Cong Xu[2], Qin Hong[2], Shutian Wang[2], Qiaoyu Zhao[2], Weihua Qiao[1], Jinkai Zang[1], Liangliang Kong[5], Fangfang Wang[5], Haikun Wang[1], Di Qu[3, 6], Dimitri Lavillette[1], Hong Tang[1], Qiang Deng[3, *], Youhua Xie[3, *], Yao Cong[2, 4, *], Zhong Huang[1, *]

[1] CAS Key Laboratory of Molecular Virology & Immunology, Institut Pasteur of Shanghai, Chinese Academy of Sciences, University of Chinese Academy of Sciences, Shanghai, China

[2] State Key Laboratory of Molecular Biology, National Center for Protein Science Shanghai, Shanghai Institute of Biochemistry and Cell Biology, Center for Excellence in Molecular Cell Science, Chinese Academy of Sciences, University of Chinese Academy of Sciences, Shanghai, China

[3] Key Laboratory of Medical Molecular Virology (MOE/NHC/CAMS), Department of Medical Microbiology and Parasitology, School of Basic Medical Sciences, Shanghai Medical College, Fudan University, Shanghai, China

[4] Shanghai Science Research Center, Chinese Academy of Sciences, Shanghai 201210, China

[5]The National Facility for Protein Science in Shanghai (NFPS), Shanghai,201210, China

[6] BSL-3 Laboratory of Fudan University, School of Basic Medical Sciences, Shanghai Medical College, Fudan University, Shanghai, China

[#] These authors contributed equally.

* Corresponding authors.

This Supplementary Information PDF includes:

Figures S1 – S12

Tables S1 – S4

a

No.	Hybridoma ID	SARS-CoV-2 RBD (OD450nm)	Receptor binding inhibition (OD450nm)	SARS-CoV RBD (OD450nm)	Pseudovirus neutralization (luminescence)
1	5A9	0.724	0.665	0.056	2455.4
2	5F4	0.446	1.57	0.07	3491.9
3	6A11	0.577	1.58	0.438	6452.6
4	6C11	0.689	0.642	0.06	4497
5	1A9	0.701	0.66	0.058	7063.6
6	1E3	0.727	1.246	0.056	4284.1
7	1E8	0.877	1.462	0.651	9719.5
8	5D2	0.673	1.196	0.078	2977.7
9	5F8	0.762	1.458	0.149	1602.4
10	6F12A	0.88	1.523	0.638	2977.5
11	6F12B	0.708	0.495	0.058	846.9
12	8A12	0.763	1.462	0.608	3635
13	8B9	0.724	1.483	0.349	6878.6
14	8B10	0.833	1.501	0.622	6971.9
15	2C2	0.81	1.46	0.623	5162.3
16	3C1	0.724	0.445	0.476	563.1
17	4C4	0.648	1.489	0.331	5658.2
18	5G2	0.679	0.941	0.055	5852.8
19	1A10	0.884	1.486	0.646	1706.3
20	5A8	0.541	1.492	0.056	3848.3
21	5B3	0.573	1.306	0.058	7095.1
22	5C1	0.91	1.437	0.689	9805.2
23	5D3	0.65	1.538	0.079	3356.9
24	5D8	0.564	1.392	0.066	2800.4
25	2H2	0.725	0.47	0.056	158.6
26	6D11	0.543	1.611	0.574	4718.2
27	2B5	0.913	1.443	0.055	4099.9
28	2G3	0.845	0.522	0.055	133.6
29	3A2	0.747	0.142	0.053	192
30	4F12	0.656	1.577	0.051	4565.7
31	8D3	0.837	0.463	0.056	246.9
ACE2-hFc only			1.602		
Pseudovirus only					8497.7

b

Assay	Number of positive clones
SARS-CoV-2 RBD binding	31
Receptor binding inhibition	9
SARS-CoV RBD binding	12
Pseudovirus neutralization	6

Supplementary Figure 1. Characteristics of 31 anti-SARS-CoV-2 hybridoma clones. **(a)** Antibody-containing culture supernatants of SARS-CoV-2 hybridomas were tested for reactivity against the RBDs of SARS-CoV-2 and of SARS-CoV by ELISA, for their ability to block the ACE2-hFc/SARS-CoV-2 RBD binding by ELISA, and for neutralization of MLV/SARS-CoV-2 pseudovirus in Vero E6 cells. For the ACE2/RBD binding-inhibition assay, bound ACE2-hFc was detected with HRP-conjugated anti-human IgG secondary antibody, and ELISA signals that decreased by 50% or more compared with the ACE2-hFc only group (OD450 value = 1.602) were considered positive for receptor binding inhibition. For pseudovirus neutralization assay (in VeroE6-hACE2 cells), luminescence value of the pseudovirus only group is 8497.7, and thus luciferase signals that decreased below 1000 are considered positive for neutralizing activity. For each assay, the positive results are highlighted in light red color. The names of 10 hybridoma clones with receptor binding inhibitory activity are colored red. **(b)** Number of positive clones for each assay is summarized in the table.

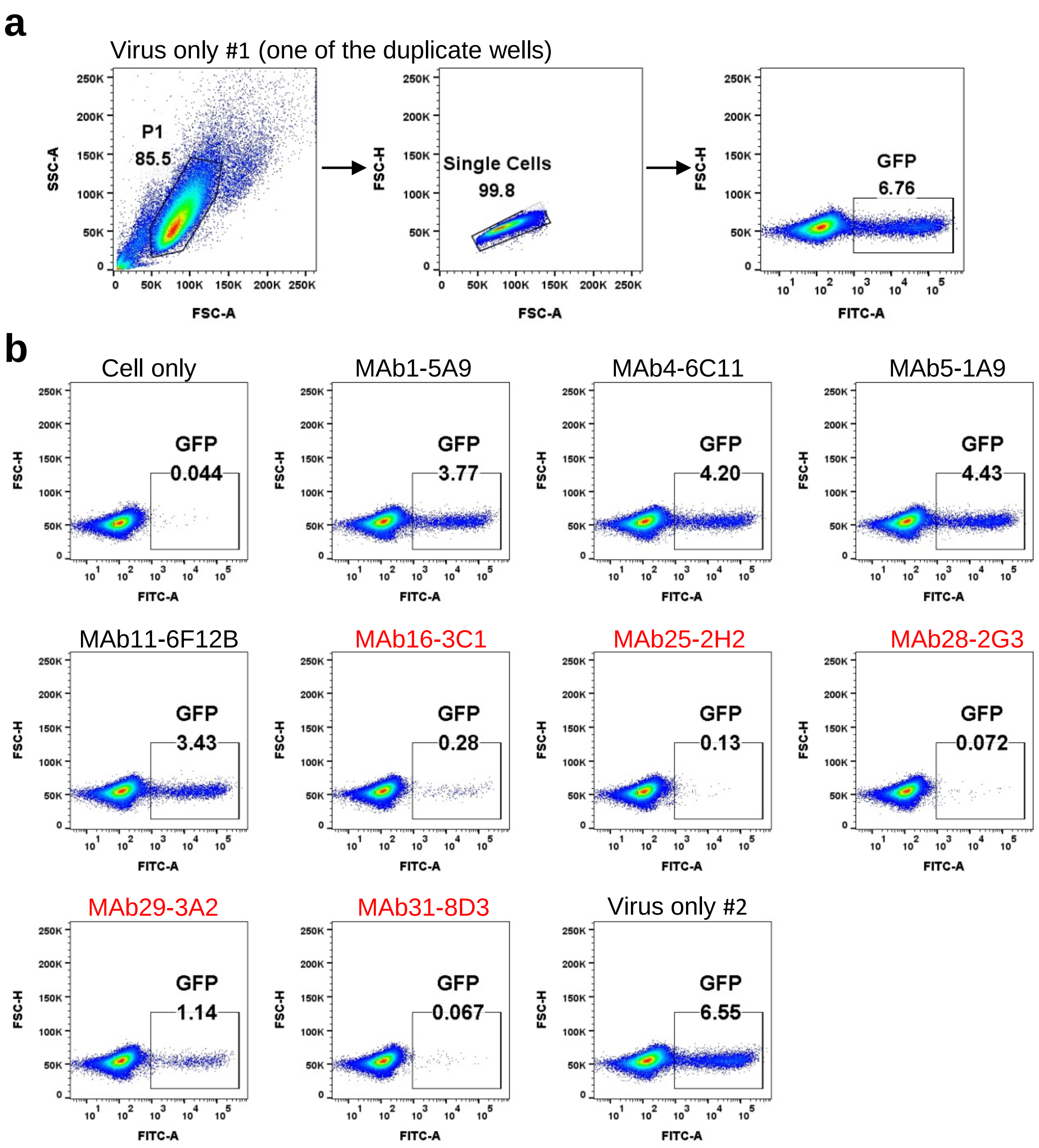

Supplementary Figure 2. Culture supernatants of the 9 hybridoma clones with receptor-binding inhibitory activity were further tested for neutralization of the GFP reporter gene-containing MLV/SARS-CoV-2 pseudovirions. **(a)** Representative FACS plots illustrating the gating strategy used in this study. GFP expression resulting from pseudovirus infection was analyzed by flow cytometry. **(b)** The percentage of GFP positive cells for each treatment was shown and a percentage less than 1.5% was considered potent neutralizing activity. The names of 5 hybridoma clones with strong neutralizing activity are colored red.

a

MAb	Heavy chain variable region				Light chain variable region		
	V	D	J	SHM (%)	V	J	SHM (%)
MAb 3C1	3-8*02	1-1*01	2*01	1.7	6-23*01	2*01	2.1
MAb 2H2	2-5-1*01	1-1*02	4*01	1	3-10*01	4*01	0.7
MAb 2G3	1-12*01	2-1*01	3*01	6.3	8-30*01	5*01	1.3
MAb 3A2	10-1*02	2-10*02	3*01	1	1-110*01	4*01	1
MAb 8D3	1-18*01	1-2*01	4*01	4.8	6-15*01	2*01	0.4

b

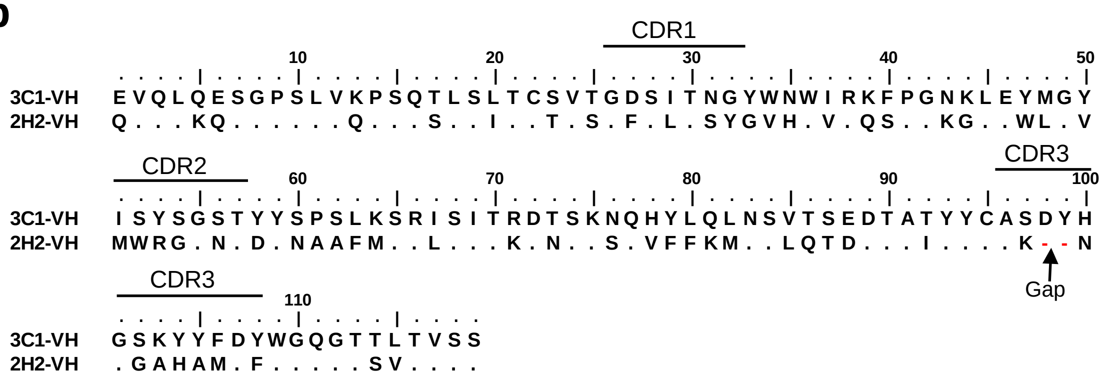

c

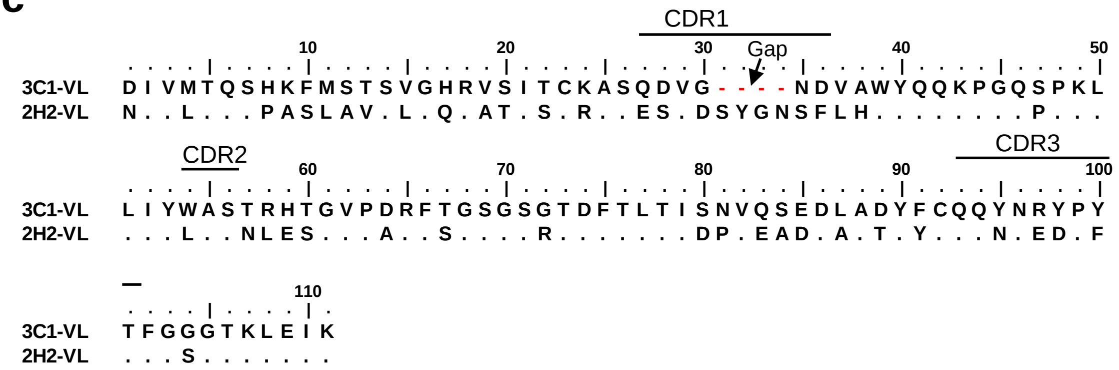

Supplementary Figure 3. Sequence analysis of the MAbs. **(a)** The closest mouse IgG germline genes were determined using IgBLAST. Somatic hypermutation (SHM) rate was calculated by comparing nucleotide sequences of variable regions of our antibodies to germline sequences. **(b-c)** Amino acid sequences of heavy-chain variable regions (V_H) (b) and light-chain variable regions (V_L) (c) of the 3C1 and 2H2 MAbs. Dots represent residues identical to those of MAb 3C1, and red dashes are gaps. Locations of complementarity determining regions (CDR) were identified by IgBLAST and indicated.

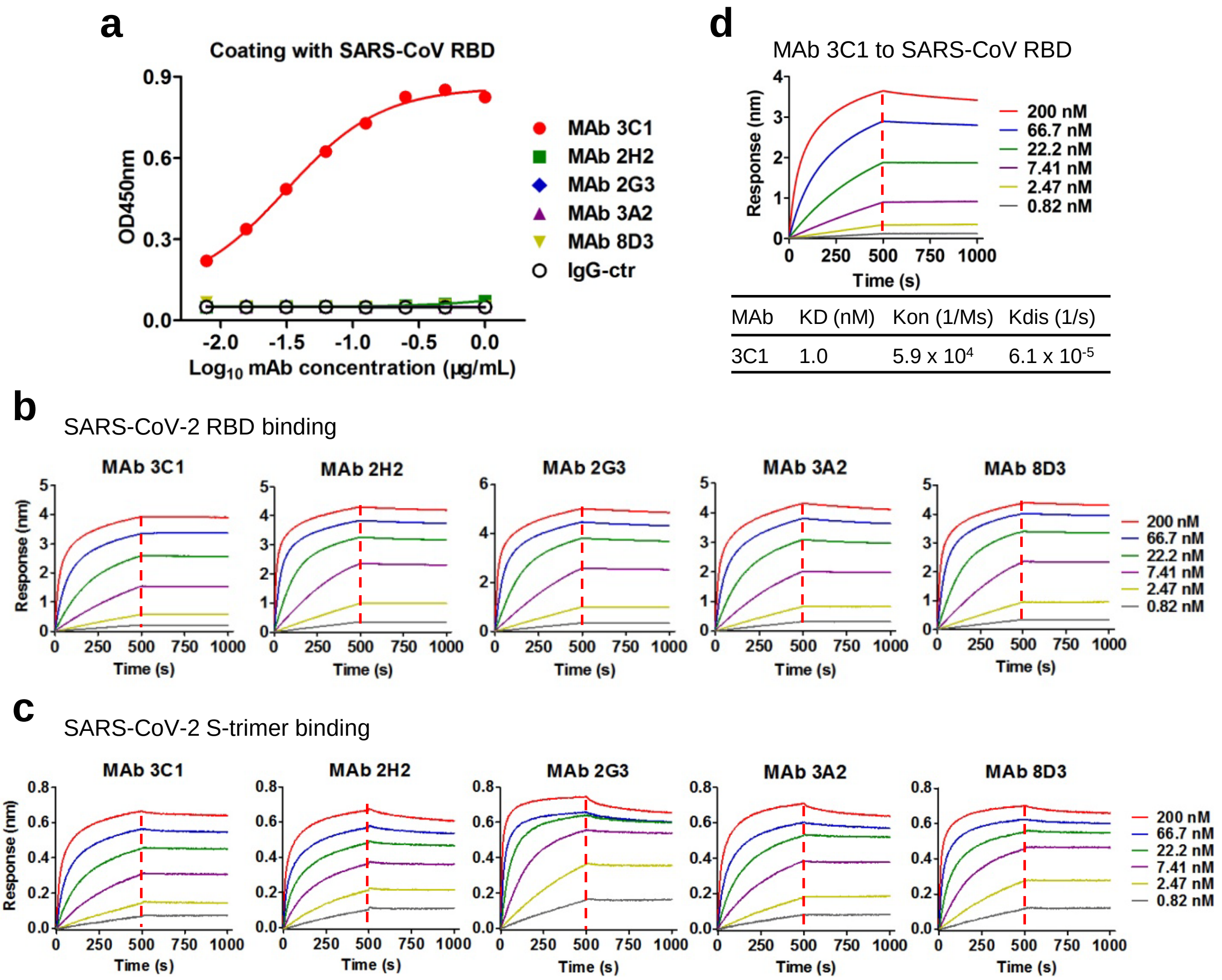

MAb	KD (nM)	Kon (1/Ms)	Kdis (1/s)
3C1	1.0	5.9×10^{4}	6.1×10^{-5}

Supplementary Figure 4. Binding properties of the MAbs (related to Figure 1). **(a)** Cross-reactivities of anti-SARS-CoV-2 MAbs to the SARS-CoV RBD measured by ELISA. Data are mean ± SEM of triplicate wells. **(b-c)** Binding kinetics of anti-SARS-CoV-2 MAbs to immobilized SARS-CoV-2 RBD (b) and S-trimer (c) measured by BLI. **(d)** Binding kinetics of MAb 3C1 to immobilized SARS-CoV RBD measured by BLI. For panels **b to d**, association and dissociation steps are divided by the dotted red line. Antibody concentrations used are shown.

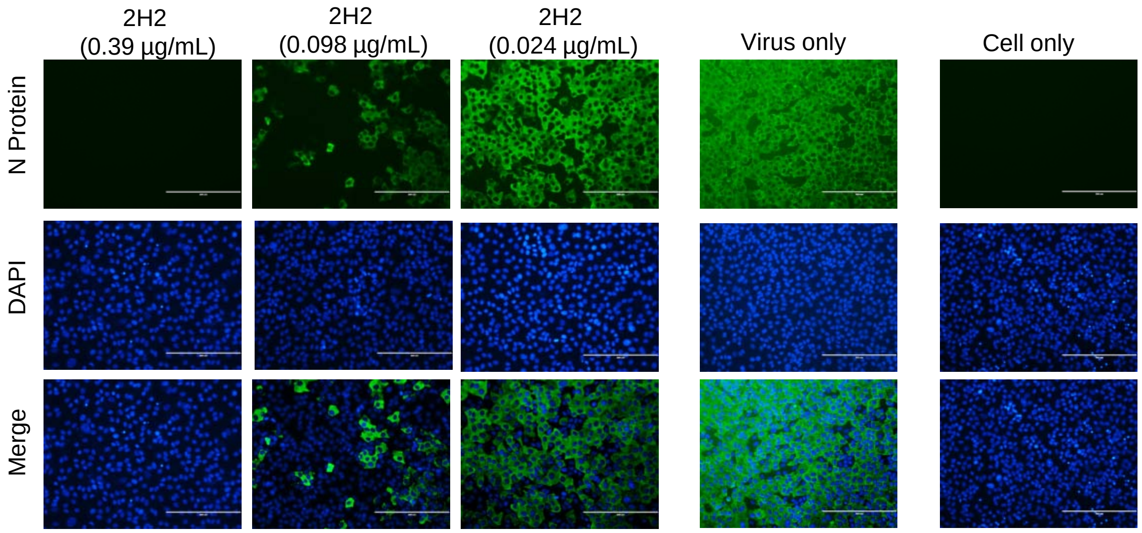

Supplementary Figure 5. Neutralization activity of the MAb 2H2 against authentic SARS-CoV-2 measured by immunofluorescence analysis. Live SARS-CoV-2 virus was incubated with serially diluted 2H2 prior to addition to VeroE6 cells. After two days, the cells were fixed and stained with N protein-specific antibody. Representative images of two independent experiments are shown. Bar, 400 µm.

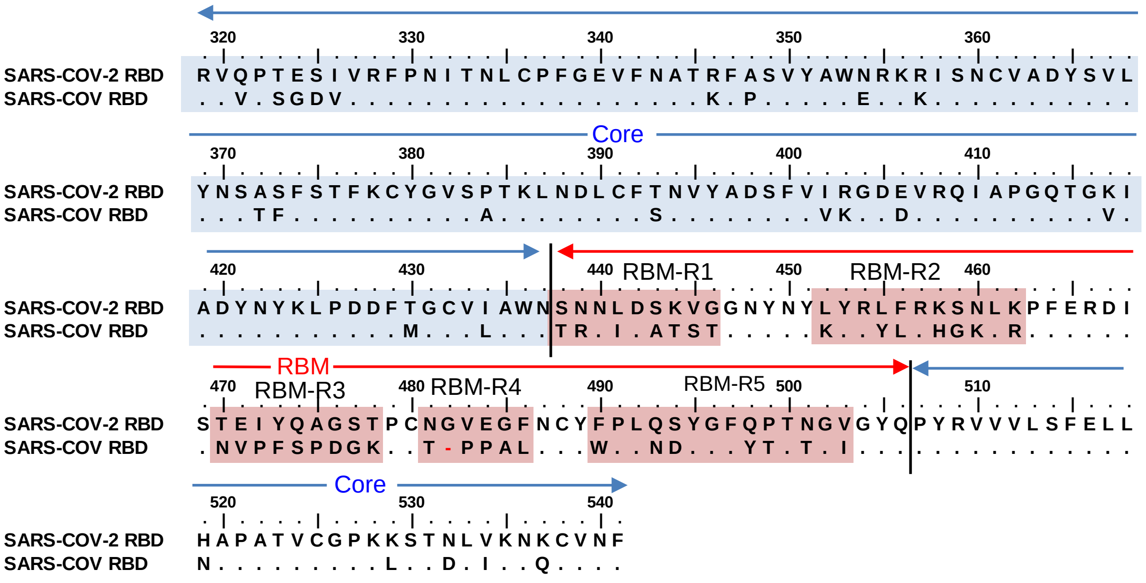

Supplementary Figure 6. Amino acid sequence alignment of RBD proteins from SARS-CoV-2 and SARS-CoV (related to Figure 2). The core and RBM regions in the RBD are indicated. N-terminal residues R319 to N437 (highlighted in light blue color) of core region in the SARS-CoV-2 RBD were mutated into the corresponding residues of SARS-CoV, resulting in cRBD (Core) mutant. The RBM has five sequence regions that exhibit considerable sequence differences between SARS-CoV-2 and SARS-CoV, and the five regions were termed RBM-R1, RBM-R2, RBM-R3, RBM-R4, and RBM-R5 and highlighted in light red color.

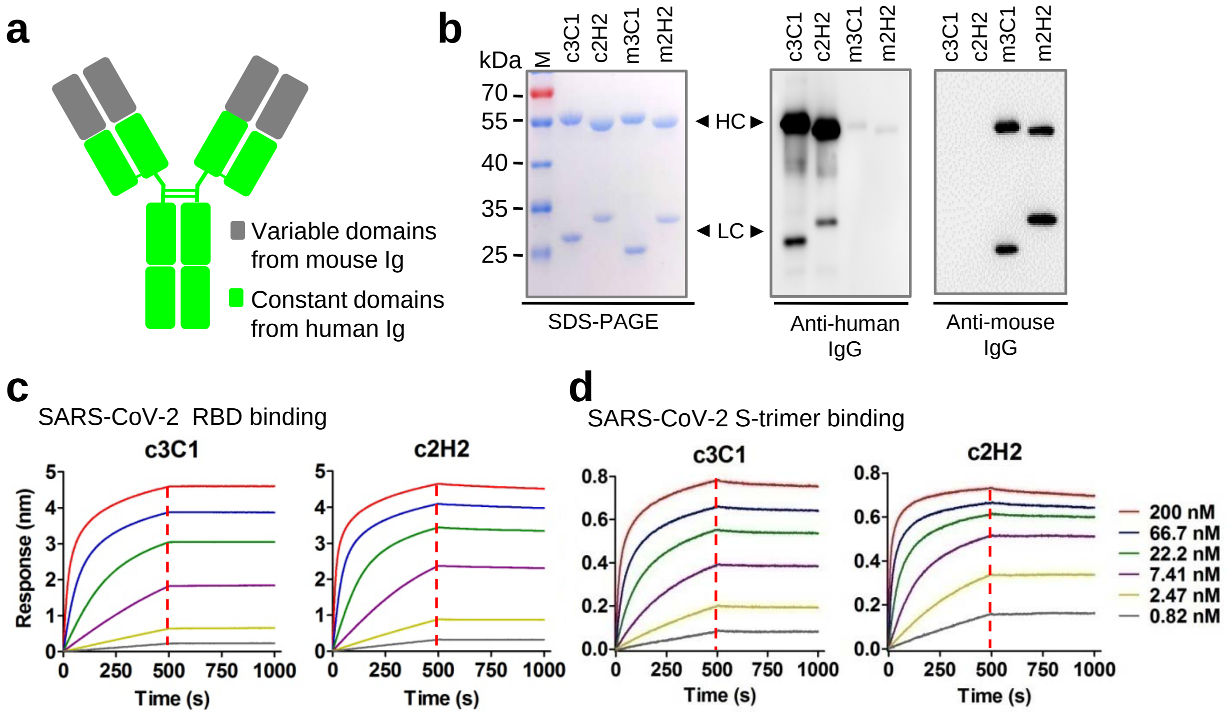

e

MAb	SARS-CoV-2 RBD binding			SARS-CoV-2 S-trimer binding			Neutralization IC50 (ng/mL) against SARS-CoV-2	
	KD (nM)	Kon (1/Ms)	Kdis (1/s)	KD (nM)	Kon (1/Ms)	Kdis (1/s)	Pseudovirus	Live virus
c3C1	< 0.001	1.2×10^5	$< 1.0 \times 10^{-7}$	< 0.001	2.0×10^5	$< 1.0 \times 10^{-7}$	758	286
c2H2	< 0.001	2.2×10^5	$< 1.0 \times 10^{-7}$	< 0.001	5.6×10^5	$< 1.0 \times 10^{-7}$	54	22
c3C1 + c2H2	Not done			Not done			27 + 27	6 + 6

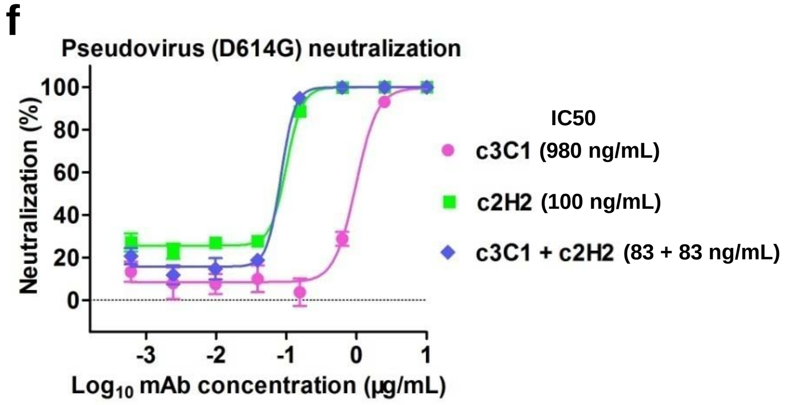

Supplementary Figure 7. Preparation and characteristics of the chimeric MAbs (related to Figure 2). **(a)** Schematic of chimeric MAb. Mouse variable domains and human constant domains are shown as gray and green boxes, respectively. **(b)** SDS-PAGE and western blotting analysis of purified chimeric MAbs. Representative images of two independent experiments are shown. Lane M, protein marker; c3C1, chimeric MAb 3C1; c2H2, chimeric MAb 2H2; m3C1, murine MAb 3C1; m2H2, murine MAb 2H2. **(c-d)** Binding kinetics of chimeric MAbs c3C1 and c2H2 to immobilized SARS-CoV-2 RBD (c) and S-trimer (d) were determined by BLI. **(e)** Summary of binding affinities and neutralization potency of the chimeric MAbs. **(f)** Neutralization activity of the chimeric antibodies against SARS-CoV-2 pseudovirus carrying the D614G mutation. c2H2 alone, c3C1 alone, and the c2H2/c3C1 (1:1) cocktail were serially diluted and assessed for neutralization of SARS-CoV-2 pseudovirus (D614G). Luciferase activity was measured two days after infection. For MAb cocktails, the concentration on the x axis is that of c2H2. Data are expressed as mean ± SEM of five replicate wells.

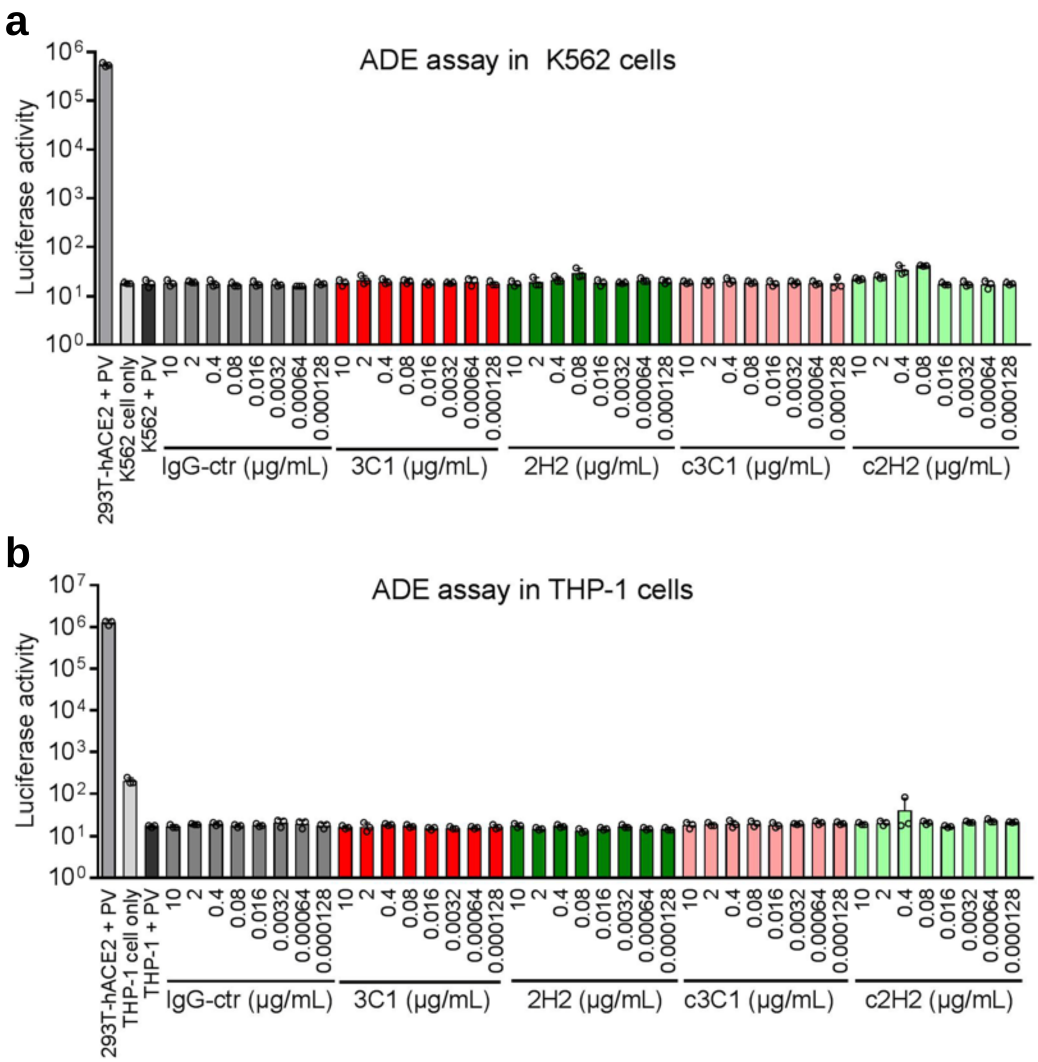

Supplementary Figure 8. MAbs 2H2 and 3C1 did not promote ADE in vitro. SARS-CoV-2 pseudovirus and serial dilutions of murine MAbs 2H2 and 3C1, chimeric MAbs c2H2 and c3C1 or isotype control antibody were incubated prior to addition to FcγR-expressing K562 cells **(a)** or THP-1 cells **(b)**. Luciferase activity was measured two days after infection. PV, pseudovirus. Data are mean ± SD of triplicate wells. Each symbol represents one well.

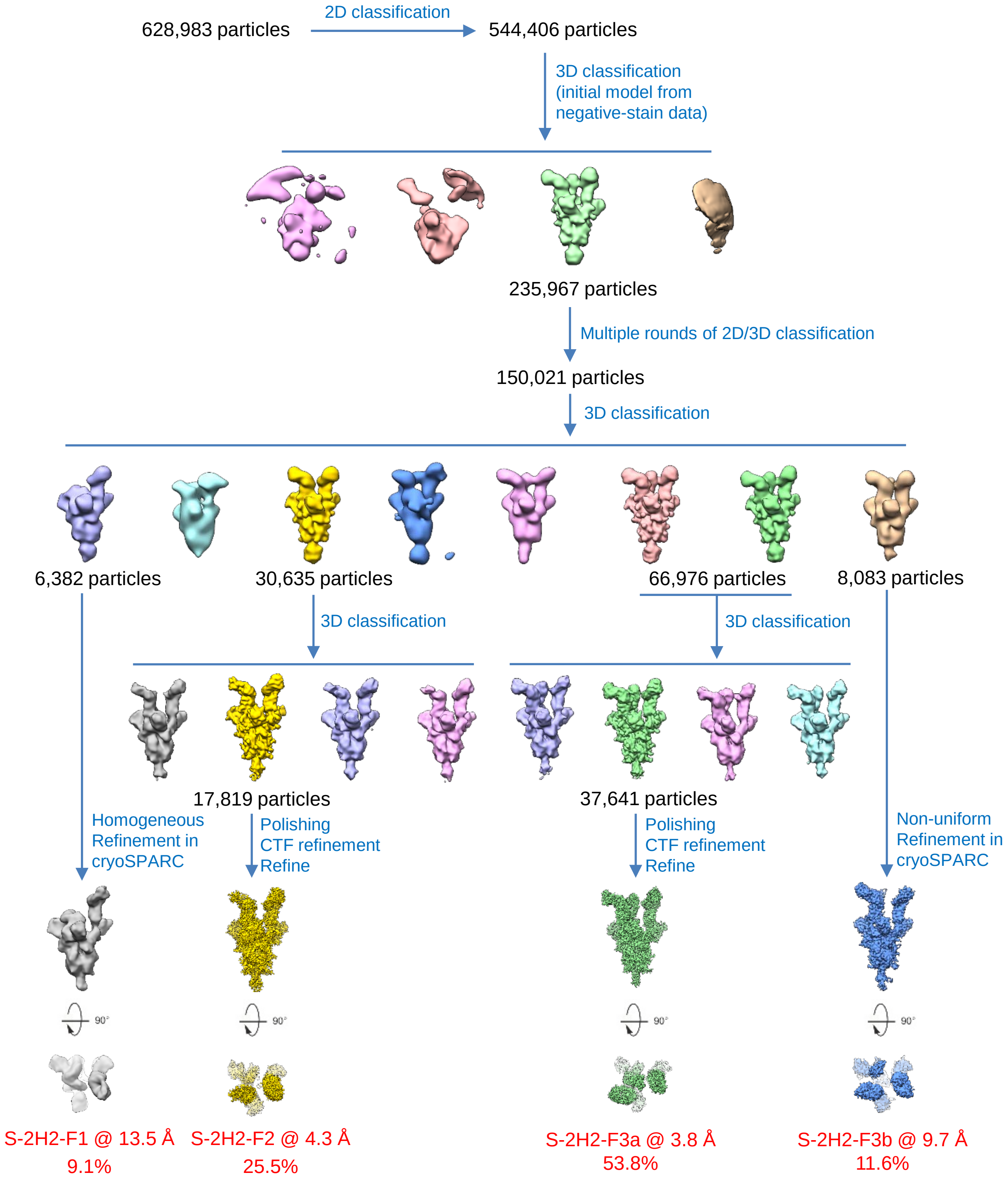

Supplementary Figure 9. Cryo-EM data processing procedure for SARS-CoV-2 S-2H2 dataset.

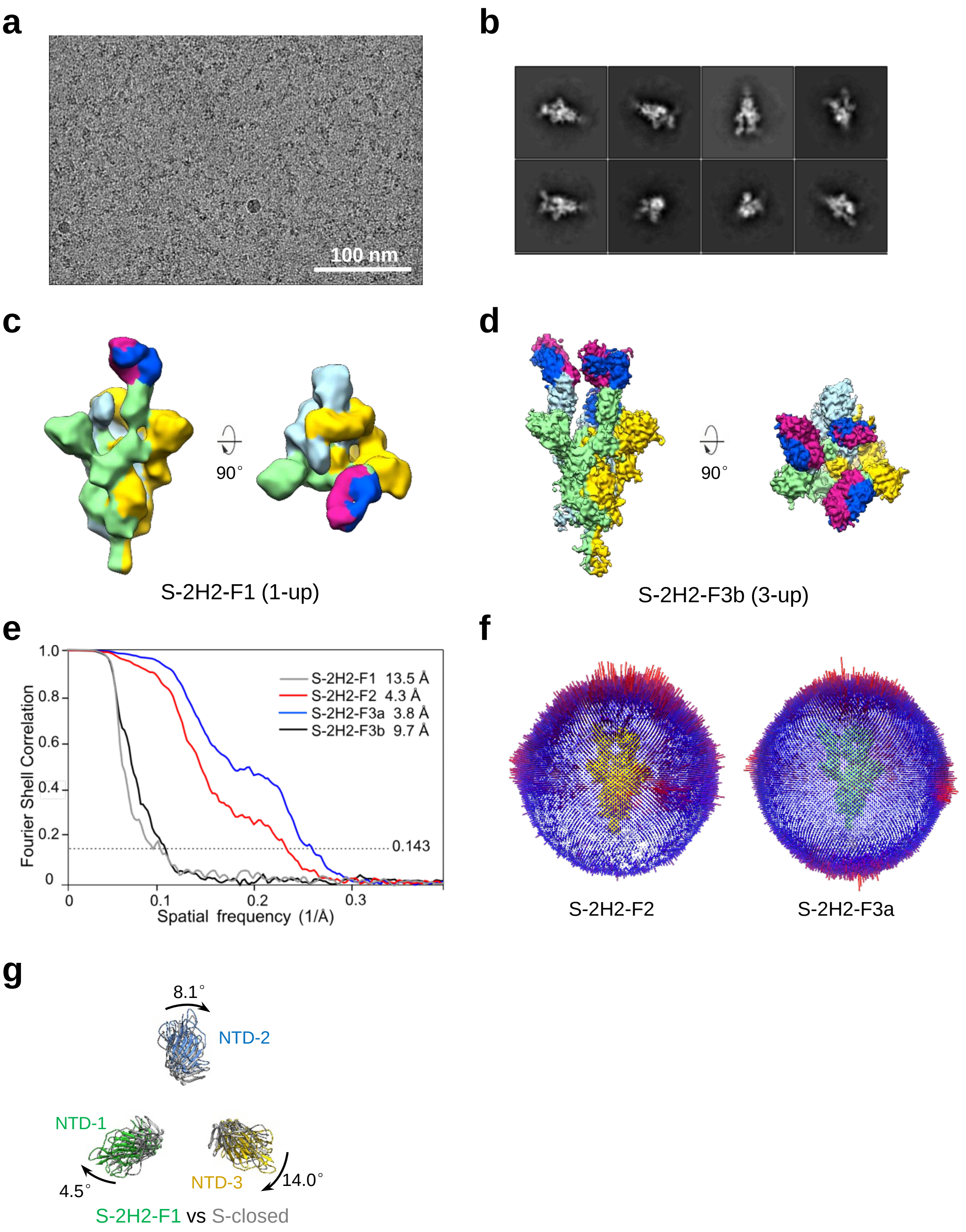

Supplementary Figure 10. Cryo-EM analysis on the SARS-CoV-2 S-2H2 complex. **(a)** A represetntaive cryo-EM image of the S/2H2 Fab complex from 6888 micrographs is shown. Bar, 100nm. **(b)** Reference-free 2D class averages of the S-2H2 complex. **(c and d)** Side and top views of the cryo-EM maps of S-2H2-F1 (c) and S-2H2-F3b (d). **(e)** Resolution assessment of the cryo-EM reconstructions by Fourier shell correlation (FSC) at 0.143 criterion. **(f)** Euler-angle distribution of the better resolved S-2H2-F2 and S-2H2-F3a maps. **(g)** Overlaid NTDs of the S-2H2-F1 (in color) with the S-close state (in grey), revealing a clockwise untwist of S-2H2-F1.

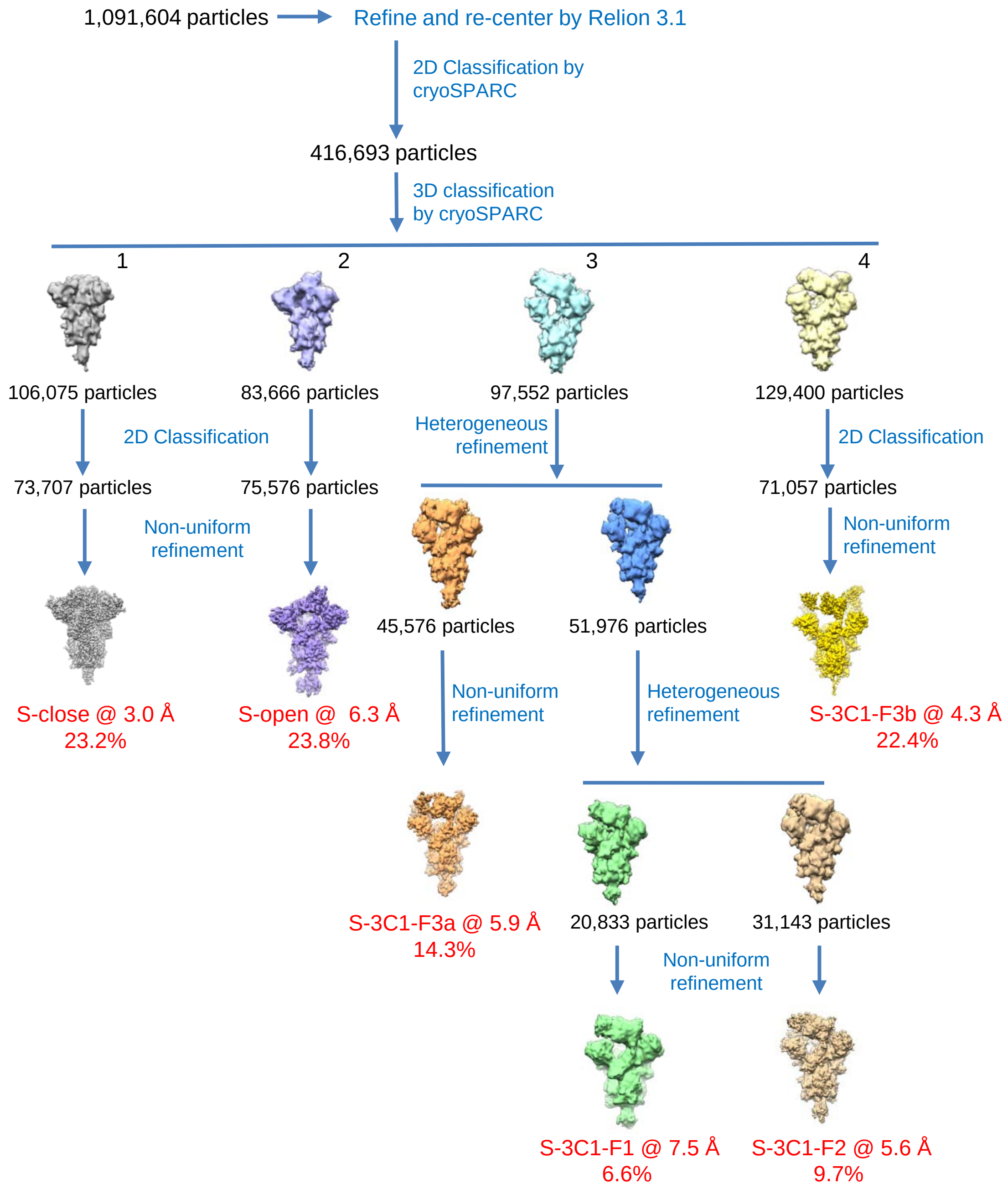

Supplementary Figure 11. Cryo-EM data processing procedure for SARS-CoV-2 S-3C1 dataset.

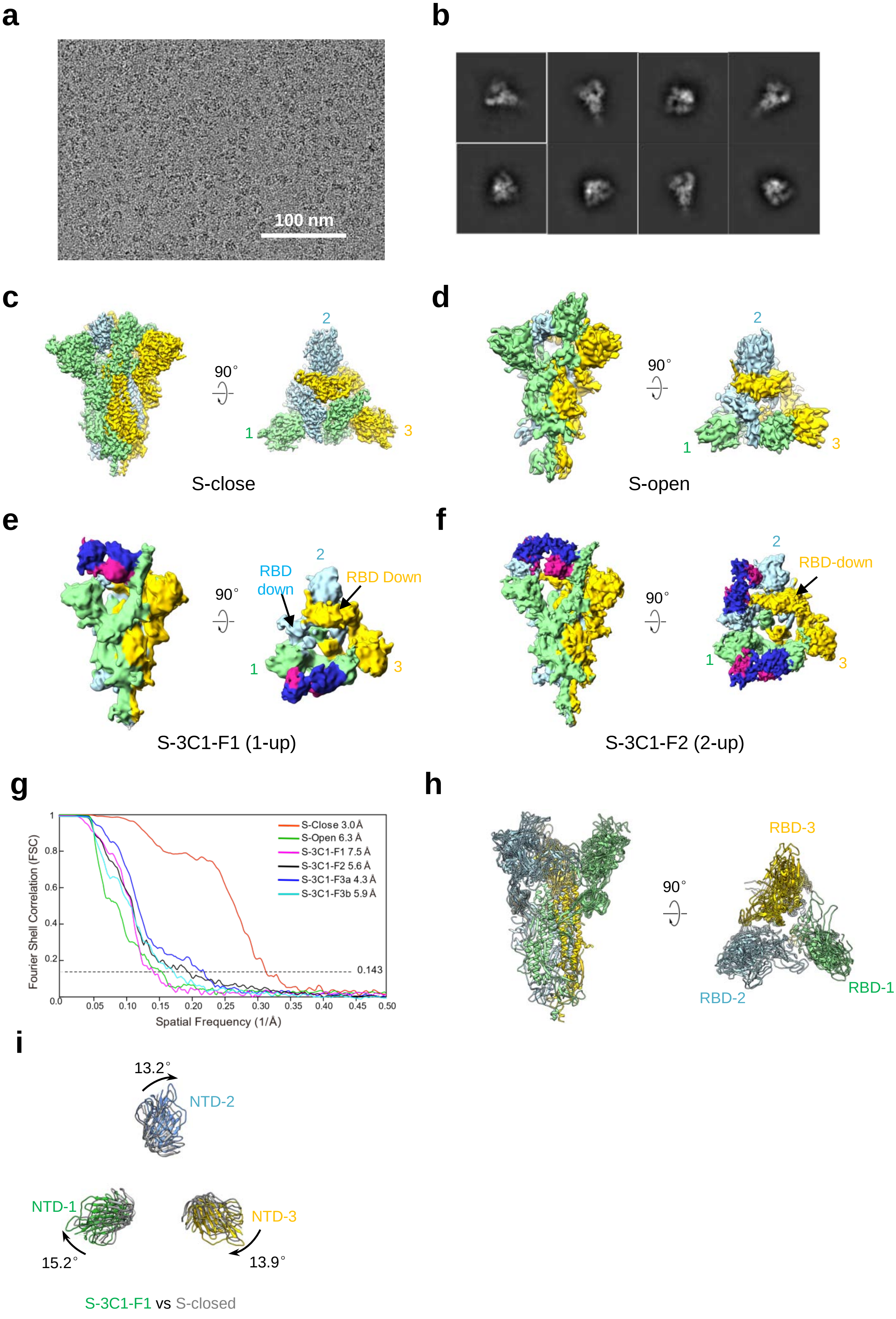
a
100 nm
b
c
90°
2
1
3
S-close
d
90°
2
1
3
S-open
e
90°
2
RBD
down
RBD Down
1
3
S-3C1-F1 (1-up)
f
90°
2
RBD-down
1
3
S-3C1-F2 (2-up)
g
Fourier Shell Correlation (FSC)
Spatial Frequency (1/Å)
S-Close 3.0Å
S-Open 6.3 Å
S-3C1-F1 7.5 Å
S-3C1-F2 5.6 Å
S-3C1-F3a 4.3 Å
S-3C1-F3b 5.9 Å
0.143
h
90°
RBD-3
RBD-2
RBD-1
i
13.2°
NTD-2
NTD-1
NTD-3
15.2°
13.9°
S-3C1-F1 vs S-closed

Supplementary Figure 12. Cryo-EM analysis on the SARS-CoV-2 S-3C1 complex. **(a)** A representative cryo-EM image of the S trimer in the presence of 3C1 Fab from 4517 micrographs is shown. Bar, 100nm. **(b)** Reference-free 2D class averages of the S-3C1 complex. **(c-f)** Side and top views of the cryo-EM maps of S-close (c), S-open (d), S-3C1-F1 (e), and S-3C1-F2 (f). **(g)** Resolution assessment of the cryo-EM reconstructions by FSC at 0.143 criterion. **(h)** Aligning the models corresponding to all the S-3C1 and S-2H2 maps together, reveals a dynamic conformational space of RBD (therefore the S1 subunit region) to coordinate the binding of 2H2/3C1 Fabs. To better render the RBD movement, the NTD was removed. **(i)** Overlaid NTDs of the S-3C1-F1 (in color) with the S-close state (in grey), revealing a clockwise untwist of S-3C1-F1.

Supplementary Table 1. Cryo-EM data collection and refinement statistics.

	S-2H2			
**Data collection**				
EM equipment	Titan Krios			
Voltage(kV)	300			
Detector	K2 Summit			
Pixel size (Å)	1.02			
Electron dose (e^{-}/Å^2)	49.6			
Exposure time (s)	6.45			
Frames	43			
Defocus range (μm)	-0.8 to -2.5			
**Reconstruction**				
Softwares	Relion 3.1 & cryoSPARC			
Structures	S-2H2-F1	S-2H2-F2	S-2H2-F3a	S-2H2-F3b
Final particles	6,382	17,819	37,641	8,083
Symmetry	C1	C1	C1	C1
Final overall resolution (Å)	13.5	4.3	3.8	9.7
**Atomic modeling**				
Softwares	Rosetta & Phenix			
Rms deviations				
Bond length (Å)	0.0039	0.0038	0.0039	0.0035
Bond Angle (°)	0.98	1.00	1.01	0.92
Ramachandranplot(%)				
Favored	96.70	96.65	97.11	96.54
Allowed	3.30	3.35	2.89	3.46
Outliers	0.00	0.00	0.00	0.00

	S-3C1					
**Data collection**						
EM equipment	Titan Krios					
Voltage(kV)	300					
Detector	K2 Summit					
Pixel size (Å)	1.02					
Electron dose (e^{-}/Å^2)	49.6					
Exposure time (s)	6.45					
Frames	43					
Defocus range (μm)	-1.0 to -2.4					
**Reconstruction**						
Softwares	Relion 3.1 & CryoSPARC					
Structures	S-close	S-open	S-3C1-F1	S-3C1-F2	S-3C1-F3a	S-3C1-F3b
Final particles	73,707	75,576	20,833	31,143	45,576	71,057
Symmetry	C3	C1	C1	C1	C1	C1
Final overall resolution (Å)	3.0	6.3	7.5	5.6	5.9	4.3
**Atomic modeling**						
Softwares	Phenix & Rosetta					
Rms deviations						
Bond length (Å)	0.0042	0.0045	0.0039	0.0044	0.0035	0.0038
Bond Angle (°)	0.97	1.04	1.02	1.04	0.89	1.00
Ramachandran plot(%)						
Favored	94.56	96.37	96.67	96.38	97.01	96.95
Allowed	5.44	3.63	3.33	3.62	2.99	2.90
Outliers	0.00	0.00	0.00	0.00	0.00	0.15

Supplementary Table 2. Contacting residues (a sidechain distance cutoff of 4 Å) at the SARS-CoV-2 RBD/2H2 interfaces

SARS-CoV-2 S RBD	2H2
R403	L58, E59, S60
D405	L58
K417*	N57
V445	Q1
G446*	Q1
Y449*	M106
Y453*	Y53
L455*	Y53, L54
F456*	Y32
A475*	D30, S31
V483	R53, G54, G55
E484	W52, R53, N98, H102
G485	W52, D58
F486*	D98, D58
N487*	N95, N96, H102
C488	H102
Y489*	S31, Y32, N95, G100, A101, H102
F490	G100
Q493*	G99, G100, D105
G496*	S60
Q498*	S60, G61
N501*	S60
Y505*	L58, E59, S60, V62, P63, A64

Heavy chain

Light chain

* ACE2 binding sites

Residues in coral indicate interactions also fulfill the criterion of < 8 Å main chain distance cutoff

Supplementary Table 3. Contacting residues (a sidechain distance cutoff of 4 Å) at the SARS-CoV-2 RBD/3C1 interfaces

SARS-CoV-2 S RBD	3C1
N501*	T30
G502*	Y53
V503	N31, G32, Y33, Y53, Y99
G504	Y33, S52, S54
Y505*	S54
Q506	N31, Y99
Y508	Y33, Y99
V433	R93
N437	Y99
A411	R93
Q414	R93
R403	S54, S56
D405	Y50, S52, S54, S56, Y58
R408	Y50, Y58, R93, Y94
Q409	Y58, R93
I410	R93
A372	N31, W50
F374	D32
S375	N92, R93
T376	N92, R93
F377	V29, N92
K378	I2, V29, Q90, N92, R93
C379	Q27
Y380	R93
S383	D28
P384	D28
T385	D28
Y369	D28, G30, G68
N370	N31

Heavy chain

Light chain

* ACE2 binding sites

Residues in coral indicate interactions also fulfill the criterion of < 8 Å main chain distance cutoff

Supplementary Table 4. A complete list of all primers used in this study

Plasmid or gene	Primer name	Primer sequence
pcDNA3.4-SARS-2-RBD (with Strep-tag)	optRBD-BamHI-F	CATCggatcctggagccacccgcagttcgaaaagggaagcgtgcagcctaccgagtcc
	optRBD-HindIII-R	CCATaagcttgcctgtgccggtcagtcc
pcDNA3.4-ACE2-hFc	ACE2-hFc-F	tcctgactggggtgagggcccagtccaccattgaggaa
	ACE2-hFc-R	ttgtcacaagatttgggctcggaaacagggggctggtt
pcDNA 3.1-SARS-2-S	Sp-F	tagcgtttaaacttaagcttatgttcgtgtttctggtgct
	Sp-685R	agatgcggagcctggggagtttgtctgggtc
	Sp-686F	actccccaggctccgcatcttctgtggcaagccagtccatc
	Sp-987R	cctcaggtgggtccagccggctcaggatat
	SP-988F	gctggacccacctgaggcagaggtgcagatcgac
	SR-R	acctcattcagcctgtcgatctccttctggatgttc
	SP1-F	gacaggctgaatgaggtggccaagaatctgaacgagtccctgatcgatctgcaggagctgggcaagtatgagcagggcagc
	SP1-R	cagcacccactcgccatccttcctcacgtaggcctggccgtccctgggggcctcagggatgtagccgctgccctgctcatacttg
	SP2-F	gatggcgagtgggtgctgctgagcaccttcctggaaaatttatattttcaaggtgattacaaggatgacgacgataagcatc
	SP2-R	tttaaacgggccctctagactcgagtcaatggtgatggtggtgatgatggtgatgcttatcgtcgtcatcc
pcDNA3.4-SARS-2-cRBD(Core)	SARS-RBD(left)-F	actggggtgagggccggatccagggtggtgccatccggc
	SARS-RBD(left)-R	attgttgctGTTCCAGGCCAGCACGCA
	nCOV-RBD(rbm) -F	tggcctggaacAGCAACAATCTGGATAGCAAAGTG
	nCOV-RBD(rbm) -R	atgatggtgatggtgaagcttGCCTGTGCCGGTCAGTCC
pcDNA3.4-SARS-2-cRBD(RBM-M2)	RBM-mut-2-F	gtatctgagacacggcaagctgaggCCATTCGAGAGGGACATCTCC
	RBM-mut-2-R	tgccgtgtctcagataccggtacttATAATTGTAGTTGCCGCCCACT
pcDNA3.4-SARS-2-cRBD(RBM-M3)	RBM-mut-3-F	ttcagcccagatggcaagCCCTGCAATGGCGTGGAG
	RBM-mut-3-R	ttgccatctgggctgaagggcacattGGAGATGTCCCTCTCGAATGG
pcDNA3.4-c3C1-hIgG1	3C1-VH-F	tcctgactggggtgagggccgaggtgcagcttcaggag
	3C1-VH-R	gatgggcccttggtgctagctgaggagactgtgagagt
pcDNA3.4-c3C1-hκ	3C1-VL-F	tcctgactggggtgagggccgacattgtgatgacccagt
	3C1-VL-R	gatggtgcagccaccgtacgttttatttccagcttggt
pcDNA3.4-c2H2-hIgG1	2H2-VH-F	tcctgactggggtgagggcccaggtgcagctgaagcag
	2H2-VH-R	gatgggcccttggtgctagctgaggagacggtgactga
pcDNA3.4-c2H2-hκ	2H2-VL-F	tcctgactggggtgagggccaacattgtgctgacccaa
	2H2-VL-R	gatggtgcagccaccgtacgttttatttccaactttgt
pShuttle-CMV-hACE2	hACE2-KpnI-F	CAACGGTACCatgtcaagctcttcctggctcct
	hACE2-XhoI-F	CCATCTCGAGctaaaaggaggtctgaacatcat
N gene (QPCR)	N-F	GGGGAACTTCTCCTGCTAGAAT
	N-R	CAGACATTTTGCTCTCAAGCTG
GAPDH gene (QPCR)	mGAPDH-F	TGCCCAGAACATCATCCCTG
	mGAPDH-R	TCAGATCCACGACGGACACA

Note that for pSecTag2A-SARS-RBD, gene synthesis and cloning was performed by GenScript (China).
