# Supplementary material for: Development and structural basis of a two-MAb cocktail for treating SARS-CoV-2 infections
Source: Nat Commun. 2021 Jan 11;12:264. doi: 10.1038/s41467-020-20465-w (PMC7801428; doi:10.1038/s41467-020-20465-w)
Supplement: Supplementary file 3 — Reporting Summary [file 41467_2020_20465_MOESM3_ESM.pdf]

nature research

Corresponding author(s): Zhong Huang

Last updated by author(s): Dec 2, 2020

Reporting Summary

Nature Research wishes to improve the reproducibility of the work that we publish. This form provides structure for consistency and transparency in reporting. For further information on Nature Research policies, see our Editorial Policies and the Editorial Policy Checklist.

Statistics

For all statistical analyses, confirm that the following items are present in the figure legend, table legend, main text, or Methods section.

n/a	Confirmed	
☐	☒	The exact sample size (*n*) for each experimental group/condition, given as a discrete number and unit of measurement
☐	☒	A statement on whether measurements were taken from distinct samples or whether the same sample was measured repeatedly
☐	☒	The statistical test(s) used AND whether they are one- or two-sided<br>*Only common tests should be described solely by name; describe more complex techniques in the Methods section.*
☒	☐	A description of all covariates tested
☒	☐	A description of any assumptions or corrections, such as tests of normality and adjustment for multiple comparisons
☐	☒	A full description of the statistical parameters including central tendency (e.g. means) or other basic estimates (e.g. regression coefficient) AND variation (e.g. standard deviation) or associated estimates of uncertainty (e.g. confidence intervals)
☐	☒	For null hypothesis testing, the test statistic (e.g. *F*, *t*, *r*) with confidence intervals, effect sizes, degrees of freedom and *P* value noted<br>*Give P values as exact values whenever suitable.*
☒	☐	For Bayesian analysis, information on the choice of priors and Markov chain Monte Carlo settings
☒	☐	For hierarchical and complex designs, identification of the appropriate level for tests and full reporting of outcomes
☒	☐	Estimates of effect sizes (e.g. Cohen's *d*, Pearson's *r*), indicating how they were calculated

Our web collection on statistics for biologists contains articles on many of the points above.

Software and code

Policy information about availability of computer code

Data collection	FEI TEM user interface 2.15.3, SerialEM 3.7.0
Data analysis	GraphPad Prism version 5, MotionCor2, RELION 3.1, EMAN2, CTFFIND 4.1.8, Phenix 1.12-2829, Rosetta 2017, COOT 0.8.7, UCSF Chimera 1.10.2, UCSF ChimeraX 1.0

For manuscripts utilizing custom algorithms or software that are central to the research but not yet described in published literature, software must be made available to editors and reviewers. We strongly encourage code deposition in a community repository (e.g. GitHub). See the Nature Research guidelines for submitting code & software for further information.

Data

Policy information about availability of data

All manuscripts must include a data availability statement. This statement should provide the following information, where applicable:

- Accession codes, unique identifiers, or web links for publicly available datasets
- A list of figures that have associated raw data
- A description of any restrictions on data availability

The authors declare that the data supporting the findings are available from the corresponding authors upon reasonable request. Cryo-EM maps determined in the SARS-CoV-2 S-2H2 dataset have been deposited at the Electron Microscopy Data Bank with accession codes EMD-30703, EMD-30704, EMD-30702, and EMD-30705, and associated atomic models have been deposited in the Protein Data Bank with accession codes 7DK5, 7DK6, 7DK4, and 7DK7 for S-2H2-F1, S-2H2-F2, S-2H2-F3a, and S-2H2-F3b, respectively. Cryo-EM maps determined in the SARS-CoV-2 S-3C1 dataset have been deposited at the Electron Microscopy Data Bank with accession code of EMD-30654, EMD-30651, EMD-30649, EMD-30642, EMD-30641 and EMD-30635, and related models have been deposited in the Protein Data Bank under accession code of 7DDN, 7DDD, 7DD8, 7DD2, 7DCX and 7DCC for S-open, S-closed, S-3C1-F1, S-3C1-F2, S-3C1-F3a and S-3C1-F3b, respectively. The sequences of 3C1-VH, 3C1-VL, 2H2-VH, and 2H2-VL have been deposited in GenBank with the accession codes MW271801, MW271802, MW271803, and MW271804,

respectively. Source data are provided with this paper.

Field-specific reporting

Please select the one below that is the best fit for your research. If you are not sure, read the appropriate sections before making your selection.

☒ Life sciences ☐ Behavioural & social sciences ☐ Ecological, evolutionary & environmental sciences

For a reference copy of the document with all sections, see nature.com/documents/nr-reporting-summary-flat.pdf

Life sciences study design

All studies must disclose on these points even when the disclosure is negative.

Sample size	For animal experiments, each group included four to seven mice. The sample size was sufficient for a good statistical analysis.
Data exclusions	No data were excluded from the analysis.
Replication	Experimental findings were reliably reproduced. Most of the experiments were replicated two or three times.
Randomization	Animals were randomly divided into experimental groups.
Blinding	Neutralization and protective activity of the MAbs against live SARS-CoV-2 was tested in a blind manner. All information about the expected outputs and the nature of the MAbs were kept from the technicians.

Reporting for specific materials, systems and methods

We require information from authors about some types of materials, experimental systems and methods used in many studies. Here, indicate whether each material, system or method listed is relevant to your study. If you are not sure if a list item applies to your research, read the appropriate section before selecting a response.

Materials & experimental systems

n/a	Involved in the study	
☐	☒	Antibodies
☐	☒	Eukaryotic cell lines
☒	☐	Palaeontology and archaeology
☐	☒	Animals and other organisms
☒	☐	Human research participants
☒	☐	Clinical data
☒	☐	Dual use research of concern

Methods

n/a	Involved in the study	
☒	☐	ChIP-seq
☐	☒	Flow cytometry
☒	☐	MRI-based neuroimaging

Antibodies

Antibodies used	Five anti-SARS-CoV-2 MAbs 3C1, 2H2, 2G3, 3A2, and 8D3 were prepared in this study; Anti-Mouse IgG (Fc specific)–Peroxidase antibody produced in goat (Sigma, catalog No. A0168-1ML, 1:10000 diluted); Rabbit Anti-Human IgG H&L (HRP) (abcam, catalog No. ab6759, 1:8000 diluted); anti-ZIKV MAb 5F8 was prepared in our lab.
Validation	Anti-SARS-CoV-2 MAbs 3C1, 2H2, 2G3, 3A2, and 8D3 were validated in this study; the specifications of commercially available antibodies can be found on the manufacture's website using their catalogue numbers. Anti-ZIKV MAb 5F8 was used isotype control and its specificity has also been demonstrated previously (Qu et al. Cell Discovery. 2020, 6:5).

Eukaryotic cell lines

Policy information about cell lines

Cell line source(s)	VeroE6 cells cells, HEK 293T cells, and SP2/0 myeloma cells, the Cell Bank of the Chinese Academy of Sciences (Shanghai, China); K562 cell line is provided from Dr. Xiaozhen Liang and was originally purchased from the American Type Culture Collection (ATCC, USA); THP-1 cell line is provided from Dr.Guangxun Meng and was originally purchased from ATCC; HEK 293F and ExpiCHO-S suspension cells, thermo fisher.
Authentication	The cell lines were not authenticated further after purchase.
Mycoplasma contamination	Cell lines have not recently been tested for mycoplasma contamination

Commonly misidentified lines (See ICLAC register)	No commonly misidentified lines were used

Animals and other organisms

Policy information about studies involving animals; ARRIVE guidelines recommended for reporting animal research

Laboratory animals	BALB/c mice. All mice (female BALB/c mice aged 6–8 weeks) were purchased from Shanghai Laboratory Animal Center (SLAC, China). The mice were kept in the SPF (specific pathogen free) animal facility with controlled temperature (20–26°C), humidity (40–70%), and lighting conditions (12 h light/12 h dark cycle).
Wild animals	No wild animals were used.
Field-collected samples	No
Ethics oversight	The animal studies were approved by the Institutional Animal Care and Use Committee at the Institut Pasteur of Shanghai.

Note that full information on the approval of the study protocol must also be provided in the manuscript.

Flow Cytometry

Plots

Confirm that:

- [x] The axis labels state the marker and fluorochrome used (e.g. CD4-FITC).
- [x] The axis scales are clearly visible. Include numbers along axes only for bottom left plot of group (a 'group' is an analysis of identical markers).
- [x] All plots are contour plots with outliers or pseudocolor plots.
- [x] A numerical value for number of cells or percentage (with statistics) is provided.

Methodology

Sample preparation	Cells were trypsinized from 24 well plates, washed with PBS, and then resuspended in staining buffer (PBS + 1% FBS + 1mM EDTA).
Instrument	A FACSCelesta flow cytometer (BD Biosciences, USA)
Software	FlowJo™ Software
Cell population abundance	No post-sort fractions were collected.
Gating strategy	Cells were first gated by FSC and SSC to obtain single cells, and then gated for GFP Positive (Infected) versus Negative (Uninfected).

- [x] Tick this box to confirm that a figure exemplifying the gating strategy is provided in the Supplementary Information.
